# Supplementary material for: Near-Complete Response to Osimertinib for Advanced Non-Small-Cell Lung Cancer in a Pretreated Patient Bearing Rare Compound Exon 20 Mutation (S768I + V774M): A Case Report
Source: Int J Mol Sci. 2024 Jul 9;25(14):7508. doi: 10.3390/ijms25147508 (PMC11277248; doi:10.3390/ijms25147508)
Supplement: Supplementary file 1 [file ijms-25-07508-s001.zip › ijms-3079945-supplementary.pdf]

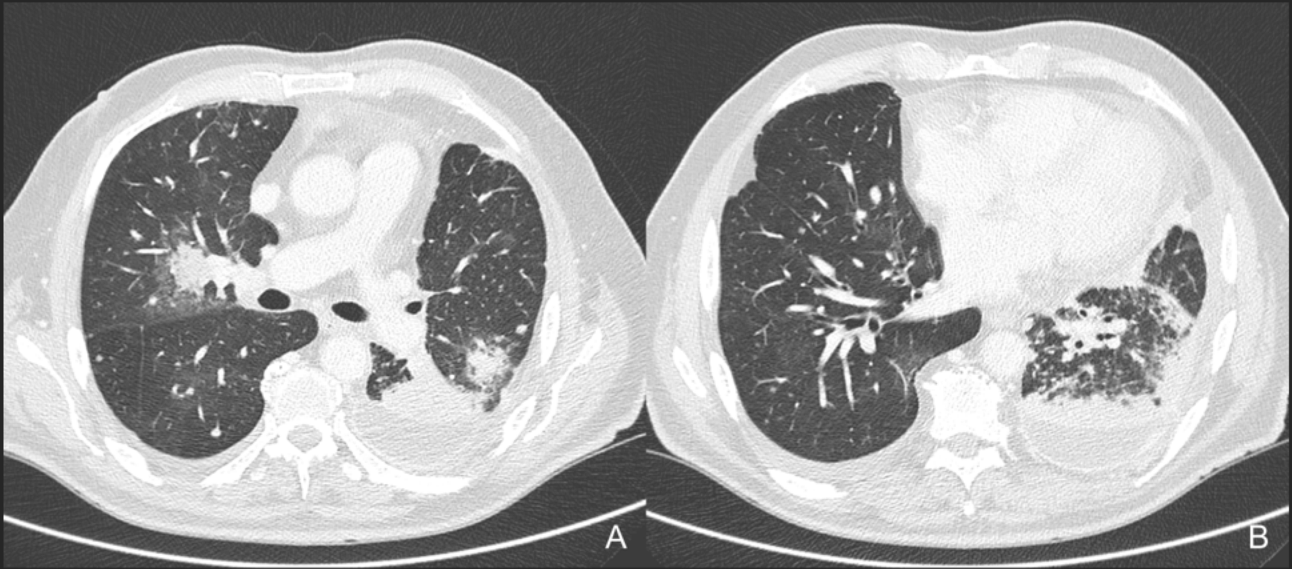

Figure S1: (CT 16/12/22): the chest CT scan, obtained after left pleurodesis, shows at the hilum (A) and base (B) levels an increase in number and size of the lung nodules. Additionally, at least two lung consolidations with ground glass halo are evident.

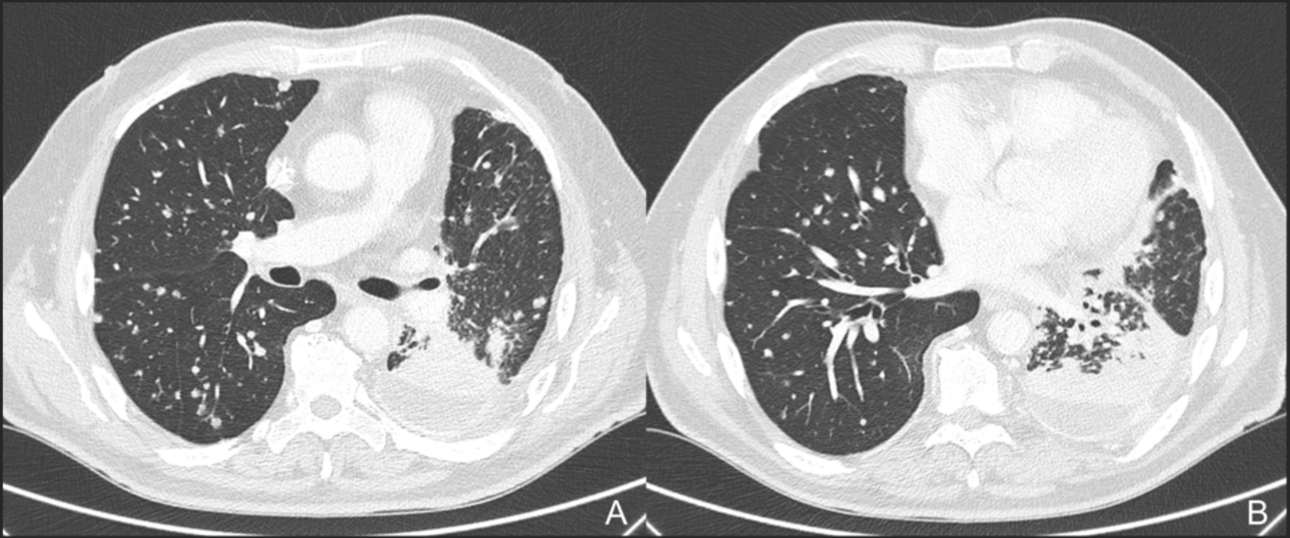

Figure S2: (CT 03/05/23): the chest CT scan demonstrates, at the hilum (A) and base (B) levels, a further increase in number and size of the lung nodules, and an increase in extent of left hilar and basal consolidations.

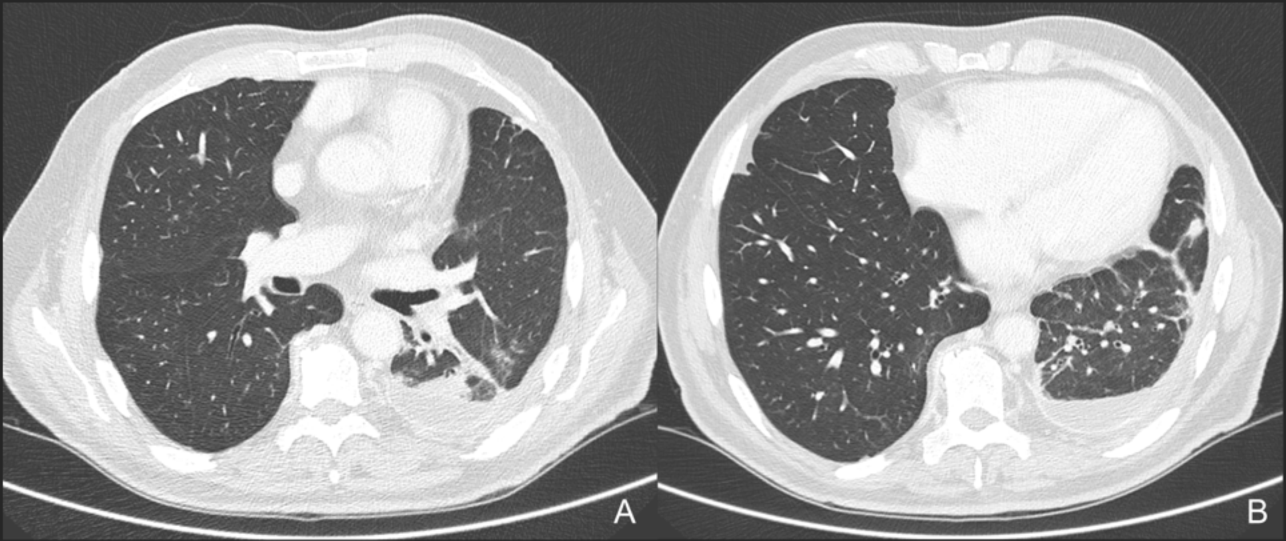

Figure S3: (CT 15/03/24): the chest CT scan shows the resolution of the lung nodules and a significant decrease in the previously evident lung consolidations at the hilum (A) and lung base (B) levels.
